# 3D Analysis of the Proximal Interphalangeal Joint Kinematics during Flexion

**DOI:** 10.1155/2013/138063

**Published:** 2013-11-05

**Authors:** Florian Hess, Philipp Fürnstahl, Luigi-Maria Gallo, Andreas Schweizer

**Affiliations:** ^1^Department of Orthopaedics, University of Zurich, Uniklinik Balgrist, Forchstrasse 340, 8008 Zurich, Switzerland; ^2^Clinic for Masticatory Disorders and Complete Dentures, University of Zurich, 8028 Zurich, Switzerland

## Abstract

*Background*. Dynamic joint motion recording combined with CT-based 3D bone and joint surface data is accepted as a helpful and precise tool to analyse joint. The purpose of this study is to demonstrate the feasibility of these techniques for quantitative motion analysis of the interphalangeal joint in 3D. *Materials and Method*. High resolution motion data was combined with an accurate 3D model of a cadaveric index finger. Three light-emitting diodes (LEDs) were used to record dynamic data, and a CT scan of the finger was done for 3D joint surface geometry. The data allowed performing quantitative evaluations such as finite helical axis (FHA) analysis, coordinate system optimization, and measurement of the joint distances in 3D. *Results*. The FHA varies by 4.9 ± 1.7° on average. On average, the rotation in adduction/abduction and internal/external rotation were 0.3 ± 0.91° and 0.1 ± 0.97°, respectively. During flexion, a translational motion between 0.06 mm and 0.73 mm was observed. *Conclusions*. The proposed technique and methods appear to be feasible for the accurate assessment and evaluation of the PIP joint motion in 3D. The presented method may help to gain additional insights for the design of prosthetic implants, rehabilitation, and new orthotic devices.

## 1. Introduction

The human finger joints with their intrinsic and extrinsic muscles perform differentiated and complex movements. Six muscle forces (extensor, deep and superficial flexor, lumbricalis, interosseous distalis, and proximalis) are involved in movements of the according joints. Traditional studies model the interphalangeal joints (proximal and distal) by simple hinge models [1–3]. However, a more current investigation [4] describes the complex incongruity of the articulating joint surfaces and the traction forces of the muscles, resulting in three-dimensional (3D) movements with several degrees of freedom. Furthermore, there is a great variance of different impacts and forces on the joint, depending on the habits of each individual. The exact knowledge of joint kinematics is the basis for developing new clinical devices such as finger joint prosthesis and orthotic tools or for improving rehabilitation of injured fingers. The impact and regulation of muscle forces and reactions on joint positions have been the subject of previous physiological studies [5–7]. Several techniques to reproduce and analyse the kinematic and kinetic properties of human joints have been described in the literature [8–10]. The results of these studies were based on interpolated data, repetitive conventional radiography, or dynamic goniometers. However, studies based on two-dimensional (2D) models may not be sufficient to analyse finger joints when facing the asymmetry of the condyles. 

Krebs et al. [11] developed a measurement technique to particularly capture small movements of the human temporomandibular joint as well as the intra-articular disc. Their method combined an accurate optical tracking system with a 3D model that was generated from medical imaging data. The goal of the herein presented study was to demonstrate that this measuring technique can be successfully applied to different anatomies in order to study physiological joint movements and changes during external impaction. Therefore, we analysed the kinematic properties of the proximal interphalangeal (PIP) joint, based on one cadaver of an index finger in a first feasibility study. 

## 2. Material and Methods

One fresh frozen human cadaveric right index finger was used in this study to reproduce the exact movements in the proximal interphalangeal joint during flexion. The used motion tracking system OPTIS [11, 12] is based on optical markers. Each marker had to be mounted to each moving part, as described in Section 2.1. An overview of the data acquisition step, that is, motion capturing and model generation, is given in Section 2.2. Finally, the quantitative evaluation of the kinematics is described in detail in Section 2.3.

### 2.1. Cadaver Preparation and Measurement Technique

**Figure 1:**
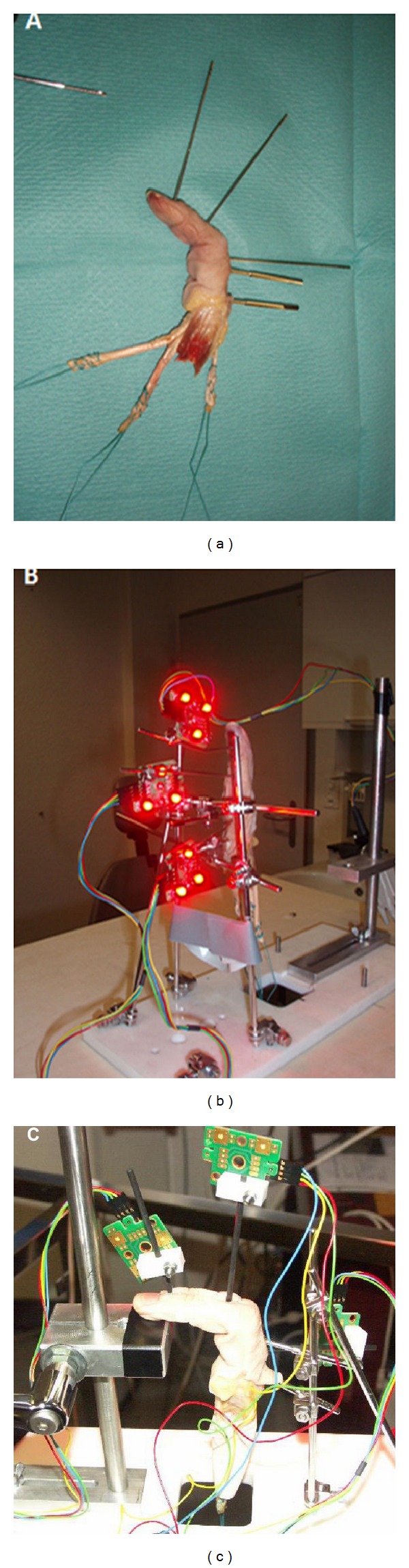
Soft tissue preparation of the index finger with dissected extensor and flexor tendons. Pins are inserted to fix the LEDs to the finger (a). The finger is fixed in a stable tool with two pins through the proximal phalangeal bone. Three cameras are detecting the light of all LEDs during flexion and extension of the finger ((b), (c)).

A complete right-hand cadaveric index finger of a 30-year-old male with intact skin, soft tissue, and extensor and flexor tendons was used. It was amputated proximally to the metacarpophalangeal joint. The extensor tendon and both flexor tendons were preserved and cut more proximally in the carpal region. At their end, a strong surgical thread was fixed which was intended to act as a pulling device for performing movements. The proximal phalanx was fixed in a stabilizing frame with two Schanz screws from the dorsal side in order to prevent rotational movements of the whole finger. The extensor tendon was split longitudinally to prevent interference with the screws during movements. Three 3 mm pins of a finger fixateur externe (AO) were bicortically inserted in the proximal, middle, and distal phalanges to ensure a stable fixing of the marker, as shown in Figure 1. Special care was taken to ensure that no interferences between the pins and tracking system did happen during movement. The motion was recorded with six degrees of freedom by means of an optoelectronic tracker, especially developed for smaller joints [12–15]. Three triangular optical markers, each carrying three LEDs, defined a proximal, medial, and distal phalangeal bone coordinate system depending, on the joint that was investigated. The target frames were fixed unilaterally to the pins. The prepared finger was fixed in a stable frame to prevent artificial movements while performing flexion.

Finally, the finger was moved to full flexion by slightly and consistently applying controlled traction to the flexor tendon. Both the superficial and profound flexor tendon were pulled equally until the full range of motion of the joint was captured. To secure a reproducible pulling force, a Newton meter was used to control the strength (Figure 1). 

### 2.2. Data Acquisition

The motion tracking system OPTIS [11, 16] was used for data acquisition. The LEDs of the markers were pulsed sequentially at 200 Hz. Three cameras were used for recording the LED positions, each equipped with a linear charge-coupled device (CCD) having 2048 elements. The device has a working volume of 20 × 20 × 20 cm, resulting in a spatial resolution below 0.05 mm [12]. The number of recorded marker positions is dependent on the acquisition time. In our case, more than 2800 positions were recorded for the flexion movement. 

For the acquisition of the bone and joint geometry, a CT scan was performed through the proximal, medial, and distal phalangeal bone with 0.65 mm slice thickness (Philips Brilliance 40 CT scanner). The whole phalangeal bone including its joint surfaces was segmented with the software application AMIRA (5.3.3, Visage Imaging GmbH, Berlin, Germany). Thereafter, 3D triangulated surface models were constructed by applying the marching cubes algorithm [17]. The CT scan was performed, including the previously fixed markers in order to determine a common reference between the tracker and CT coordinate system. Each marker contained metallic spheres whose positions were known in the tracker coordinate system. Since the sphere centres had been also identified in the CT scan, the resulting point correspondences were used to determine the coordinate transformation by solving the absolute orientation problem. 

### 2.3. Data Analysis

The tracking system provided a 3D position for each of the three LEDs of the medial phalangeal bone's marker relative to its reference marker in the proximal phalangeal bone. In our experiment, over 1800 positions were recorded. Based on this set of 3D points, the relative motion of the medial phalangeal bone from its initial position to full flexion was calculated. In each time step, the 4 × 4 transformation matrix *T*_*i*_ was obtained by solving the absolute orientation problem [18] for the three corresponding 3D point pairs in step 0 and step *i*. Thereafter, we applied three different 3D quantification methods to the data.

The finite helical axis (FHA) analysis is a well-accepted method for joint movement quantification and visualization. In this approach, the relative transformation between two positions of a body is described by a finite rotation around and a translation along the FHA. The angle of rotation *θ*_*ij*_, the directional vector *n*_*ij*_, and the displacement can be calculated by the relative transformation matrix *T*_*ij*_ = *T*_*i*_^−1^∘*T*_*j*_ for consecutive positions *i* and *j*, using Rodriguez's formula:

(1) $$\begin{array}{c} \theta_{ij}=\cos^{-1}\left(\frac{\mathrm{tr}\left(R\right)-1}{2}\right),\;\;n_{ij}=\frac{r_{1}\times r_{2}}{||r_{1}\times r_{2}||}, \end{array}$$

where tr⁡(*R*) is the trace of the 3 × 3 orientation matrix *R* of *T*_*ij*_ and *r*_*k*_ is the *k*th row of *R*.

Lastly, the positional vector of the FHA is calculated as described in [19].

Since the FHA is ill defined if *θ*_*ij*_ approaches to zero, the initial data was sampled in 1 degree steps. The total rotation around the FHA during flexion is represented by the angle *θ*_0*n*_, where *n* is the position in full active flexion. The angular variation of the FHA was obtained by measuring the angular difference between the first axis *n*_01_ and *n*_*ij*_, for all *i* > 0. 

In addition to the helical axis analysis, we focused on the determination of a fixed coordinate system that best describes the motion of the phalangeal bone with respect to the flexion axis. To this end, we followed a numerical optimization approach. Let *C* be the coordinate system with unknown axes and unknown centres. The transformation *T*_*i*_ can be expressed with respect to *C* as a transformation matrix *T*_*i*_′ = *C* ^−1^∘*T*_*i*_. Further, *T*_*i*_′ can be decomposed in a rotational part expressed as three Euler angles *φ*_1_, *φ*_2_, and *φ*_3_ and a translation vector *t*′. Finally, *C* can be determined by minimizing

(2) $$\begin{array}{c} \underset{i}{\min}\left(f_{i,}g_{i}\right),\;\text{where}\;f_{i}=||t^{\prime }||,\;\;g_{i}=\left|\varphi_{2}\right|+\left|\varphi_{3}\right|. \end{array}$$

For the initial guess of the coordinate system, the centre was set to the 3D centre of the condyles of the proximal bone. The initial axes were equal to the Cartesian coordinate axes. 

The goal of the optimization step was to primarily describe the motion as a rotation around a single axis (*φ*_1_) while minimizing the translation and the rotation around the remaining axes (target functions *f*_*i*_ and *g*_*i*_). As a consequence, *t*′ and (*φ*_2_, *φ*_3_) can be interpreted as the translational and rotational errors if the joint motion would be simplified to a hinge.

In the last step of the kinematic analysis, the articular distance of the PIP joint was measured during flexion. To this end, the closest 3D point in the model of the fixed reference bone was determined for each 3D point of the moving bone model. The distances were tracked during flexion and stored along with each surface point, resulting in a high-resolution distance field over time. This distance field was analysed and visualized with respect to the flexion angle. A KD-tree was applied for closest point queries in order to reduce the computational time.

## 3. Results

### 3.1. FHA Analysis

**Figure 2:**
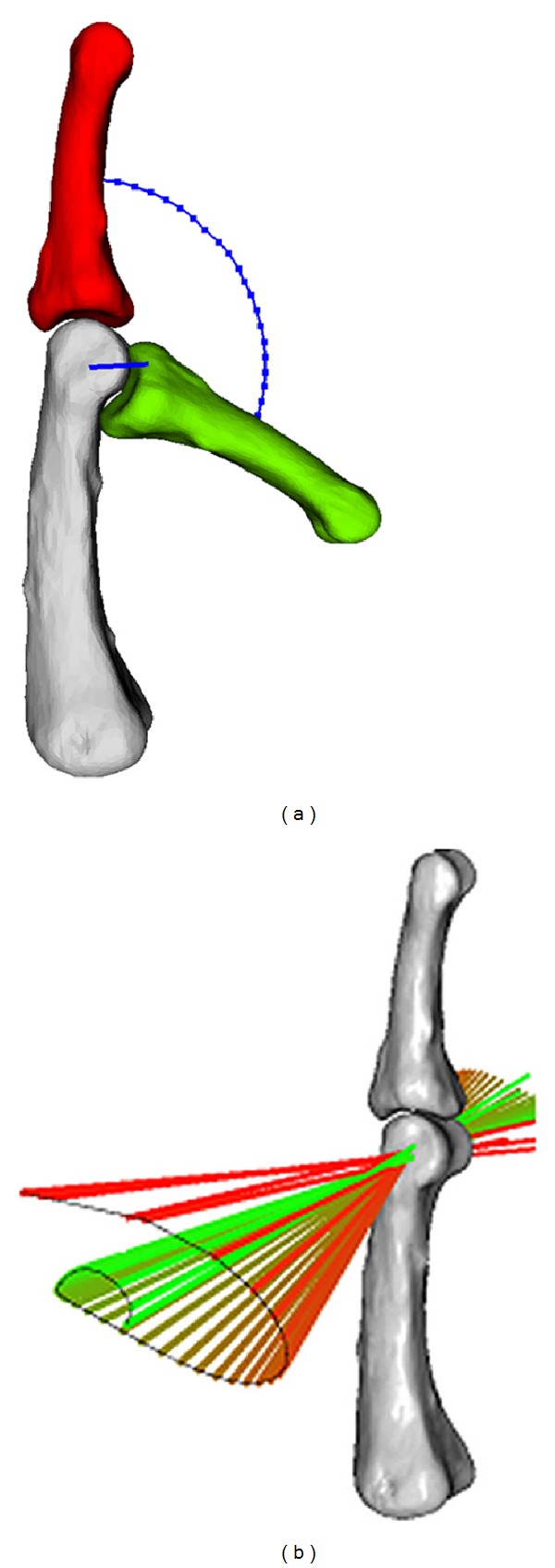
(a) Starting and ending position of the PIP joint motion from extension (red) to flexion (green). The motion trajectory (blue curve) and average screw axis (blue line) are given. (b) Motion of the PIP joint from neutral position (red model) to full flexion (green model) described by a sequence of finite helical axes (from red to green). The axes are defined relative to the first FHA shown in red. The last FHA (full flexion) is denoted in green.

**Figure 3:**
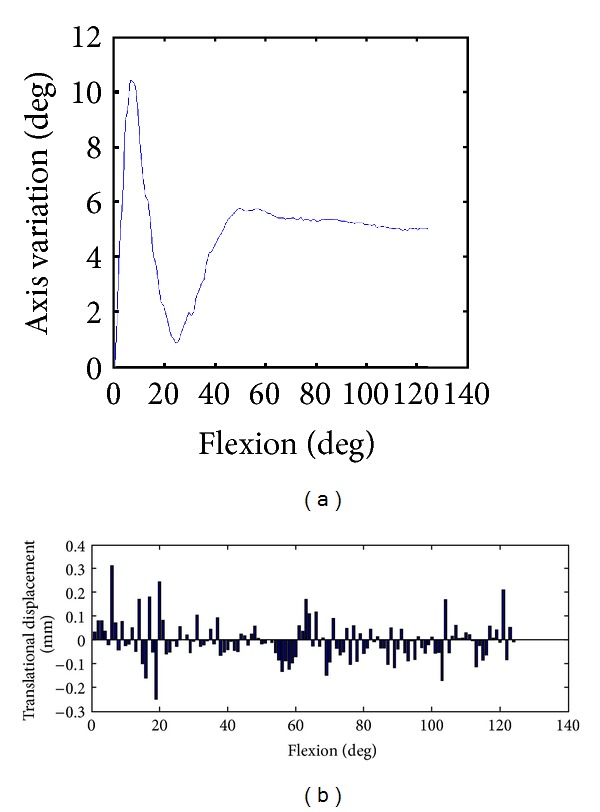
(a) FHA variation during flexion. The angular variation of the axis with respect to the degree of flexion is given in degrees. (b) Normalized translational displacement along the axis (pitch) is given in mm with respect to the degree of flexion.

The overall rotation *θ*_0*n*_ around the FHA from neutral position to full active flexion was 123.9°. The visualization of the ruled surface, derived from the FHA, has a spiral-like shape, as shown in Figure 2(b). The FHA varied by up to 10.5° (4.9 ± 1.7° on average), as shown in Figure 3(a). While the main variation of the axis direction was observed between 0° and 50° of flexion, the direction did not vary significantly above 50° of flexion. The translation along the axis between a one-degree step was always below 0.31 mm, as demonstrated in Figure 3(b).

### 3.2. Coordinate System Analysis

**Figure 4:**
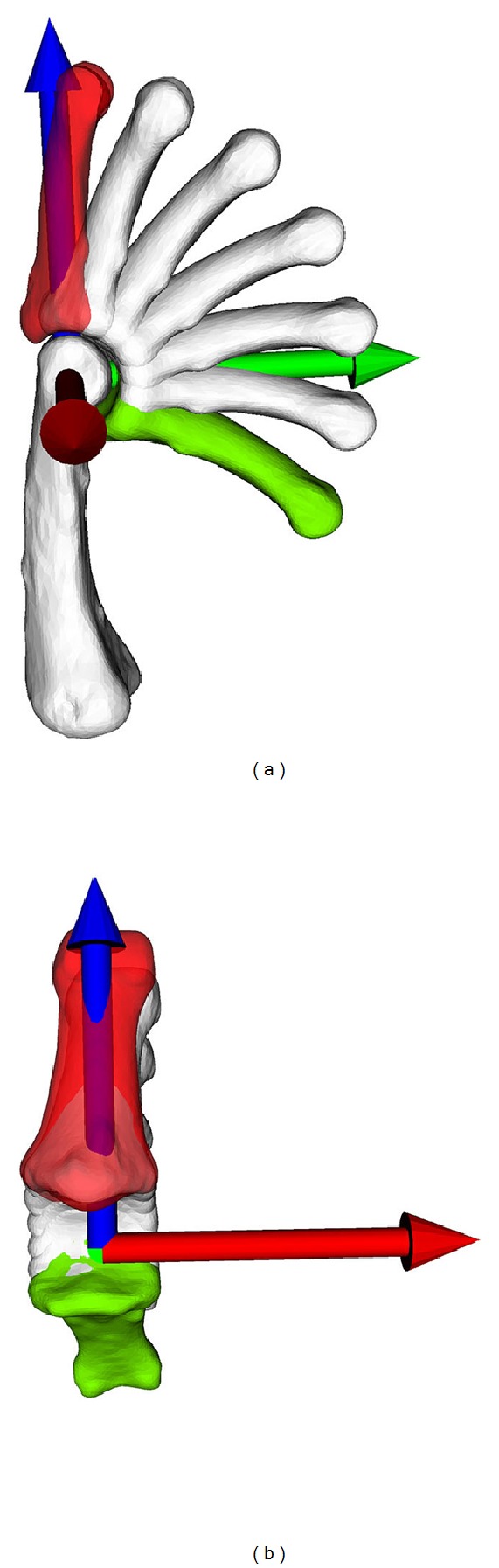
Location of the calculated coordinate system from a sagittal view (a) and anterior-posterior view (b). (a) Palmar is to the right, and ulnar direction is towards the camera. (b) Ulnar is to the right.

**Figure 5:**
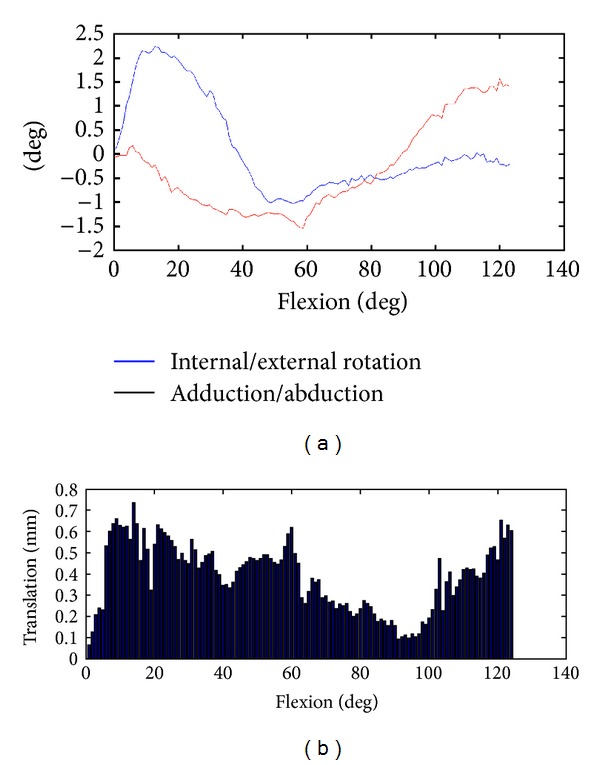
(a) Rotation during flexion with respect to the fixed coordinate system. Rotation around adduction/abduction (red line) and internal/external (blue line) is given. (b) Translation in mm during flexion with respect to the fixed coordinate system.

**Figure 6:**
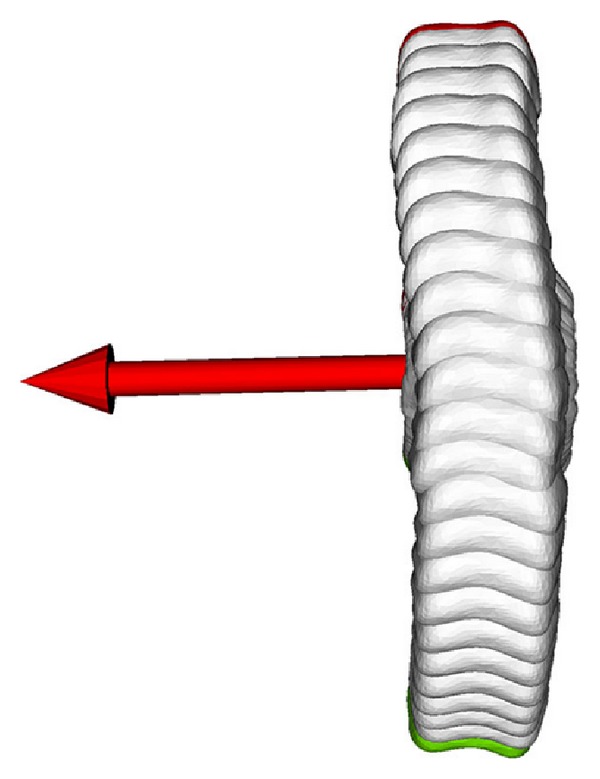
Motion of the PIP joint from neutral position (red model, top) to full flexion (green model, bottom) viewed from a distal position. The flexion axis (red) is pointing to ulnar direction.

The optimized origin of the fixed coordinate system *C* was located in the centre of the condyles of the proximal phalangeal bone in the sagittal plane, as depicted in Figure 4(a). However, in anterior-posterior view, the origin was not the centre of the condyles; it was located towards the ulnar (Figure 4(b)). The measured rotation around the axis being regarded as the flexion axis was 124.0°. The rotation around the other two axes, namely, adduction/abduction and internal/external rotation, is given in Figure 5(a). In adduction/abduction, the rotation ranged between −1.5° and 1.5° (−0.3 ± 0.91° on average). The measured angle in internal/external rotation ranged from −1.1° to 2.2° (0.1 ± 0.97° on average). A small translation between 0.06 mm and 0.73 mm was observed throughout the motion, as depicted in Figure 5(b). These small translational and rotational deviations can be visualized best by expressing the motion from a distal point of view, as demonstrated in Figure 6.

### 3.3. Intra-articular Joint Distance Analysis

**Figure 7:**
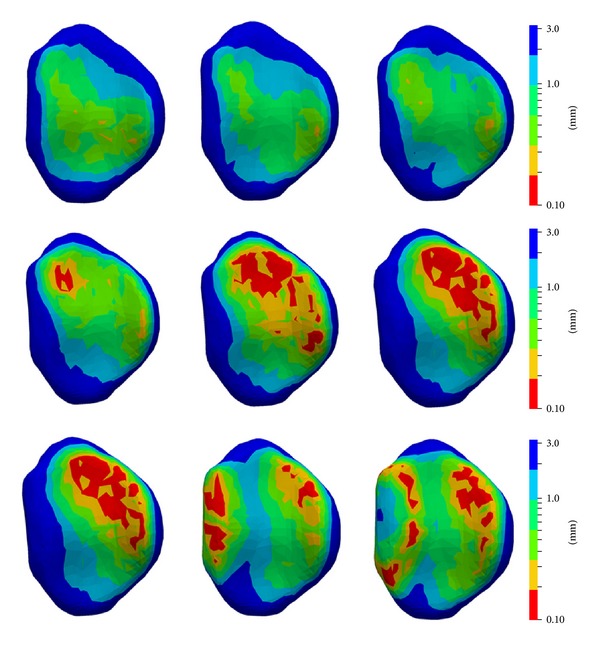
Visualization of the intra-articular distance during flexion, depicted in steps of 15°. Palmar is to the left and radial is to the top. The distance is shown in a range from 0 to 3 mm, using a logarithmic scale. First row (left to right): articular distance between neutral position and 30°. Second row (left to right): articular distance between 45° and 75°. Third row (left to right): articular distance between 90° and full flexion.

Figure 7 shows the evaluation of the articular distance during flexion movement, depicted in 15° steps. Until 30° of flexion, the minimal distance measured was above 0.3 mm. Subsequently, the minimal distance decreased to less than 0.1 mm in positions above 30° of flexion. The contact area was primarily located at dorsal and radial (see middle row of Figure 7). In full flexion, an additional contact was observed in the palmar part of the joint.

## 4. Discussion 

The aim of this study was to show that the described tracking device can be used for the evaluation of bones which are different to dental anatomy. We successfully demonstrated its application on the PIP joint of a right index finger. 

Motion recording in combination with CT data had been successfully applied to various anatomies [10]. However, previous work targeting PIP kinematics did not report such combined approaches. In Leijnse et al. [20], a kinematic evaluation of the finger's interphalangeal joint was described, using a precise optical camera system. Van Sint Jan and colleagues [21] reported a CT-based method that determines the motion between subsequent CT scans by using anatomical landmarks. Other approaches were based on 2D kinematic models that are explained with the help of devices [22] or replicas [7]. Several biomechanical models of the finger are available as well [23]. Most of them describe the PIP motion as a hinge joint. 

The presented work aimed to demonstrate the capabilities of a precise experimental and computational technique and show how the simulation can help to understand the kinematics of physiological finger joint movements. The combination of 3D data from a CT scan and the kinematic data from the camera system allowed not only an exact 3D analysis of joint position during flexion but also an evaluation of the interacting joint surfaces in 3D.

For a quantitative evaluation of the PIP joint kinematics, three different methods were presented and applied. We were able to derive a ruled surface from the FHA movement at the PIP joint which has a spiral-like shape, largely more complex than a 2D hinge movement. Therefore, incongruity in curvature of the two articulating surfaces [7], rotational and slight dorso-palmar translational components could lead to the movement of the helical axis. 

Furthermore, a mathematical optimization approach was presented that expressed the motion using a fixed coordinate system. Small rotational movements up to 2.5° in adduction/abduction and in internal/external rotations as well as translations of up to 0.5 mm were observed. In radioulnar direction, the bone performed an S-shaped motion with a general tendency towards the ulnar (see Figure 6). This tendency may also be the reason why the origin of the fixed coordinate system was not centred but biased in ulnar direction. 

The spiral motion shown here might be a part of the physilogical motion of the PIP joint. Together with the movement of the MP and DIP joints, the typical Fibonacci curve in the sagittal plane is well known. The spiral motion characterises the movements of the finger joints in a frontal/coronal plane and may be part of the physiological course of motion.

The 3D evaluation and visualization of the distance within the PIP joint may be a valuable tool for making assumptions about weight bearing. During flexion, the areas of contact shift to the dorso-radial side, as it can be also observed in Figure 2. This dorsal translation is also described in [7]. In full flexion, an additional contact was measured proximally. This can be explained by the fact that the proximal border of the middle phalangeal bone is in close proximity to the proximal bone in full flexion (green model in Figure 2).

Understanding the PIP joint as a complex 3D articulation may have relevant clinical impacts. Morphological, geometrical, and dynamical characteristics of the interphalangeal joint are important in the treatment approaches of finger joint arthroplasty. Futher, open reduction and internal fixation of intraarticular fractures are demanding and premise the knowledge of the physiological morphology and kinematic. Also, the understanding of the 3D kinematics has potential value in the design of improved orthotic devices after trauma and/or surgery for more accurate rehabilitation. The presented work has the limitation that only one specimen was used for the PIP joint analysis. Additionally, finger movements were provoked only by traction of the deep and superficial flexor tendon and the extensor tendon. In physiological conditions, always a certain cocontraction of the extensor tendon is apparent that is, however, dependent on flexion movement against resistance. In this pilot setting, we aimed to record the movement with as low as possible joint forces. The influences of the lumbricals and interossei muscles were ignored in this study. The here described techniques and methods have the potential to help quantifying and analysing the motion of this complex joint. Whether they can be used to predict extreme impactions on joint movements of the human finger in vivo must be proven in a larger study. This is subjected to further research.
